# An extremely rare case of neuromuscular and vascular hamartoma of the appendix

**DOI:** 10.1186/s40792-020-00970-2

**Published:** 2020-08-24

**Authors:** Takahiro Sasaki, Tomohisa Furuhata, Masashige Nishimura, Tatsunori Ono, Akiyoshi Noda, Hirotaka Koizumi, Nobuyoshi Miyajima, Takehito Otsubo

**Affiliations:** 1 Digestive Disease Center, Toyoko Hospital, St. Marianna University School of Medicine, 3-435, Kosugicho, Nakahara-ku, Kawasaki, Japan; 2 Department of Pathology, Toyoko Hospital, St. Marianna University School of Medicine, 3-435, Kosugicho, Nakahara-ku, Kawasaki, Japan; 3 Department of Gastroenterological and General Surgery, St. Marianna University School of Medicine, 2-16-1, Sugao, Miyamae-ku, Kasawaki, Japan

**Keywords:** Appendix, Neuromuscular and vascular hamartoma

## Abstract

**Background:**

Neuromuscular and vascular hamartoma is a rare lesion of the small intestine, with only 26 cases reported since its initial description in 1982. No occurrence of hamartoma in the appendix has been reported until now.

**Case presentation:**

A 60-year-old man had been suffering from longstanding right lower quadrant pain. Abdominal computed tomography showed a slight swelling of the appendix as the possible cause of his pain. Laparoscopic appendectomy with partial resection of the cecum was performed for diagnostic and therapeutic purposes. An 18 × 10-mm lesion located on the tip of the appendix was found in the resected specimen. Pathological examination showed that the lesion was covered with normal mucosa and consisted of adipose tissue, smooth muscle fibers, small vessels, and neural fibers. These findings were consistent with neuromuscular and vascular hamartoma of the appendix.

**Conclusion:**

This is the first report of neuromuscular and vascular hamartoma arising from the appendix.

## Background

Neuromuscular and vascular hamartoma (NMVH) is a rare lesion of the small intestine, often clinically presenting as non-specific abdominal pain, recurrence of obstructive symptoms, and gastrointestinal bleeding. First described by Fernando and McGovern in 1982, this lesion consists of an aberrant proliferation of neural, muscular, and vascular elements in the small intestine [1]. Only 26 cases have been reported since its initial description, and NMVH occurred in the small intestine in all cases except one, in which it was found in the cecum [1–20]. To our knowledge, NMVH occurring in the appendix has not been reported in the English literature until now. We report the first case of NMVH of the appendix.

## Case presentation

**Fig. 1:**
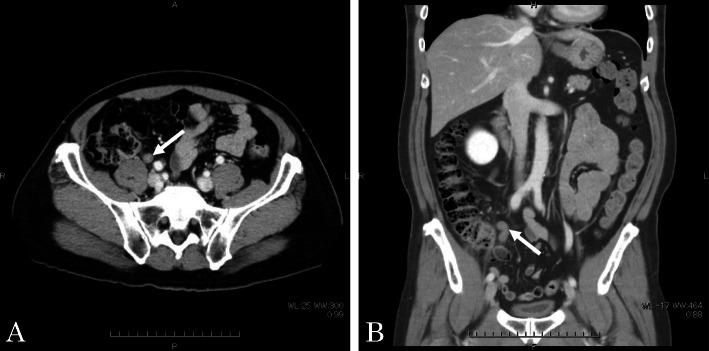
Abdominal computed tomography (**a**: axial, **b**: sagittal). Appendix is slightly swollen (arrow) without obvious neoplastic tumors or lymphadenopathy

**Fig. 2:**
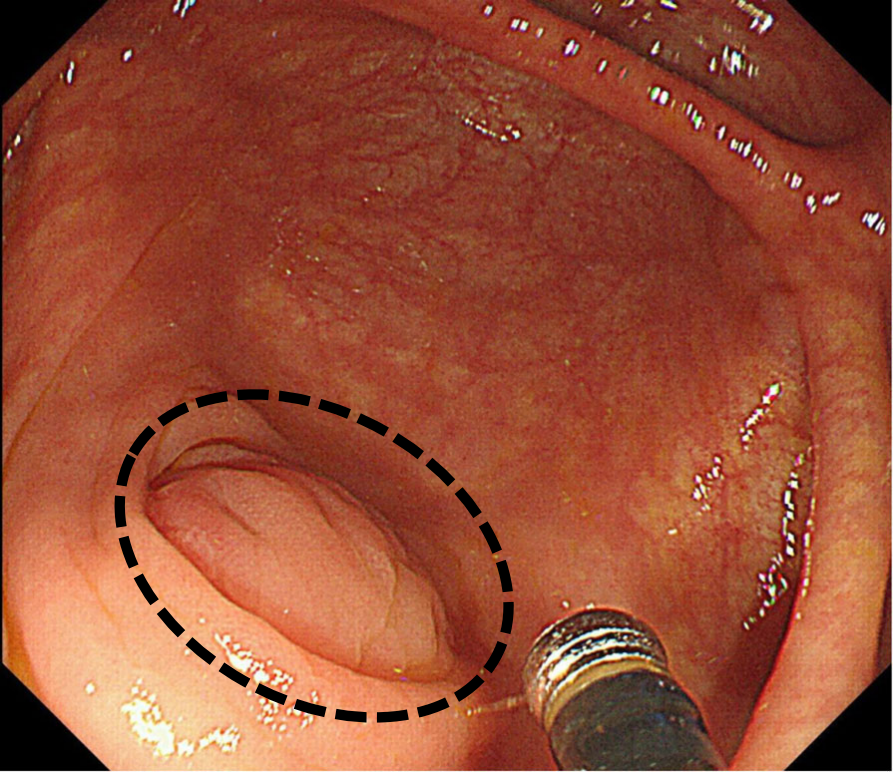
Colonoscopy. An elevated mucosal lesion is observed around the appendiceal orifice (circle)

**Fig. 3:**
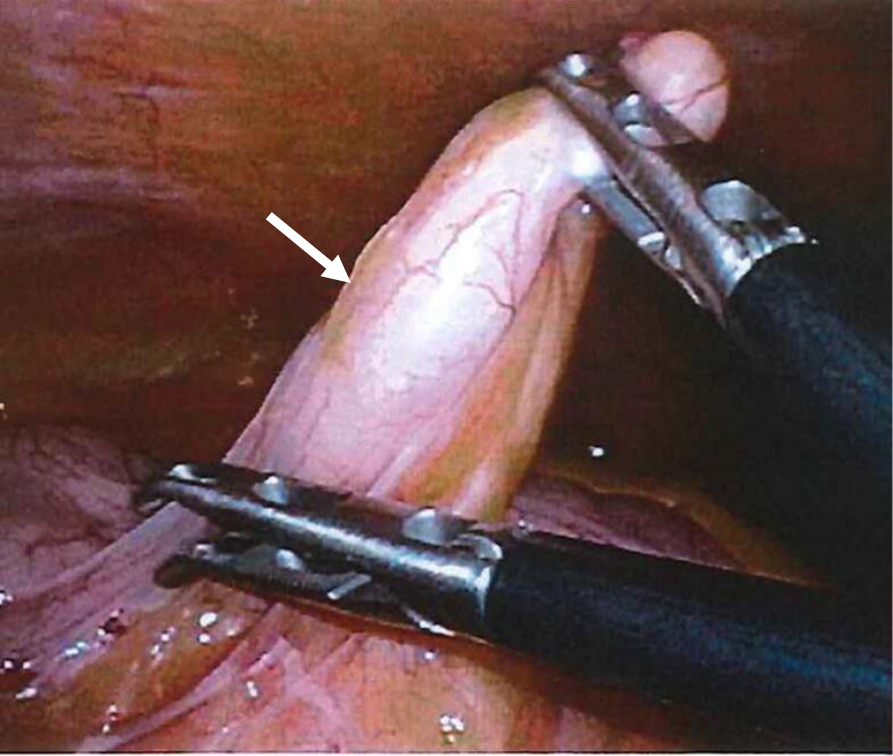
Laparoscopic appendectomy. Intraoperative findings were a slight swelling of the appendix (arrow), but no other findings on the serosal surface of the appendix and cecum on exposure of the tumor

**Fig. 4:**
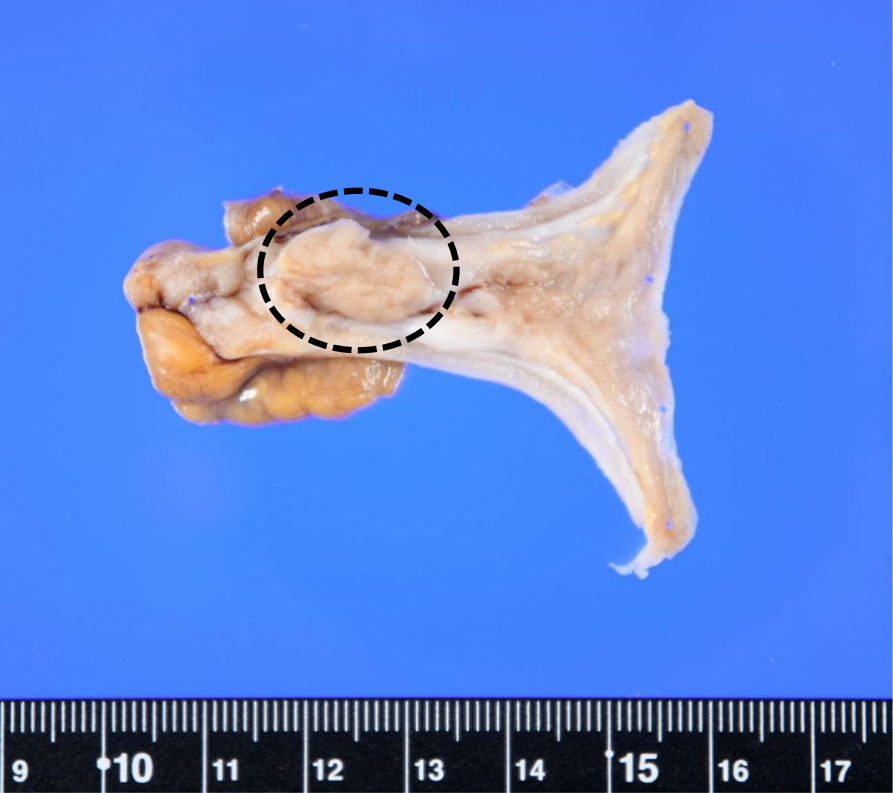
Surgical specimen. A submucosal lesion measuring 18 × 10 mm (circle) is present on the tip of the appendix

**Fig. 5:**
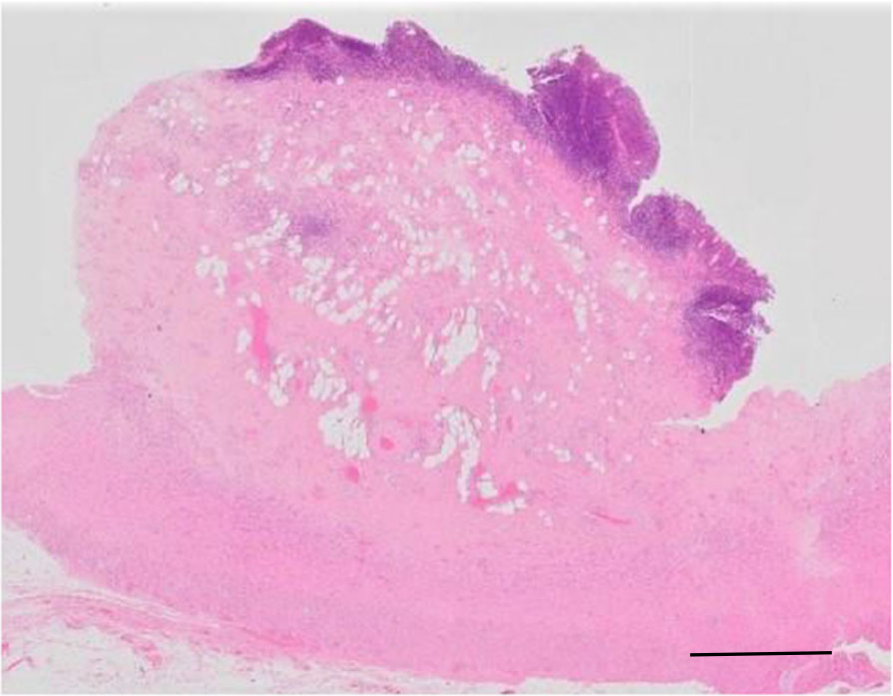
Hematoxylin and eosin staining (Loupe image). The submucosal lesion contained abundant adipose tissue and vascular vessels. Bar = 2 mm

**Fig. 6:**
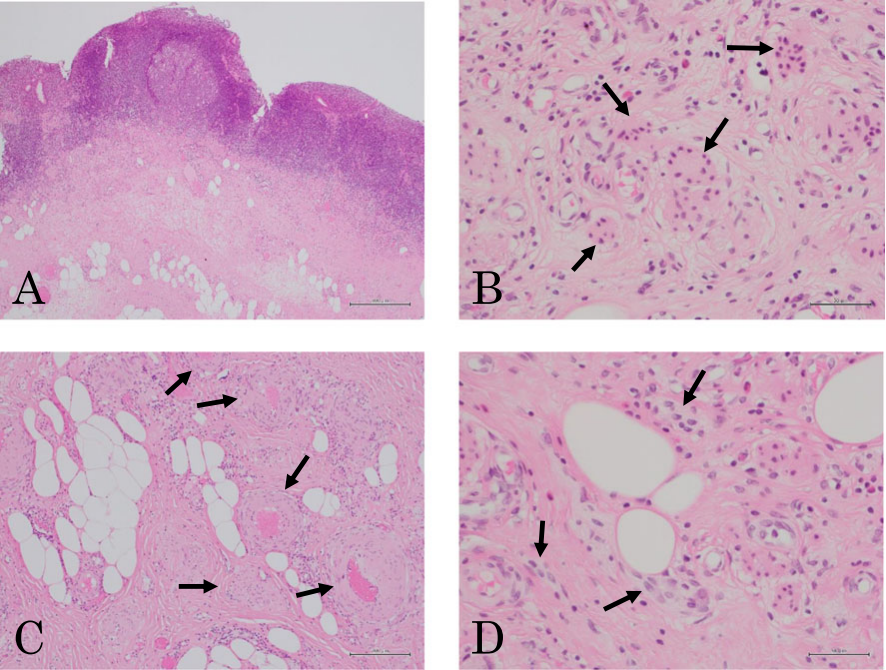
Hematoxylin and eosin staining. The submucosal lesion was covered with normal mucosa (**a**) and consisted of adipose tissue (**a**, **c**, **d**), smooth muscle fibers (**b**, arrows), small vessels (**c**, arrows), and neural fibers (**d**, arrows). Original magnification, **a**: × 40; **b**, **d**: × 200; **c**: × 100

**Fig. 7:**
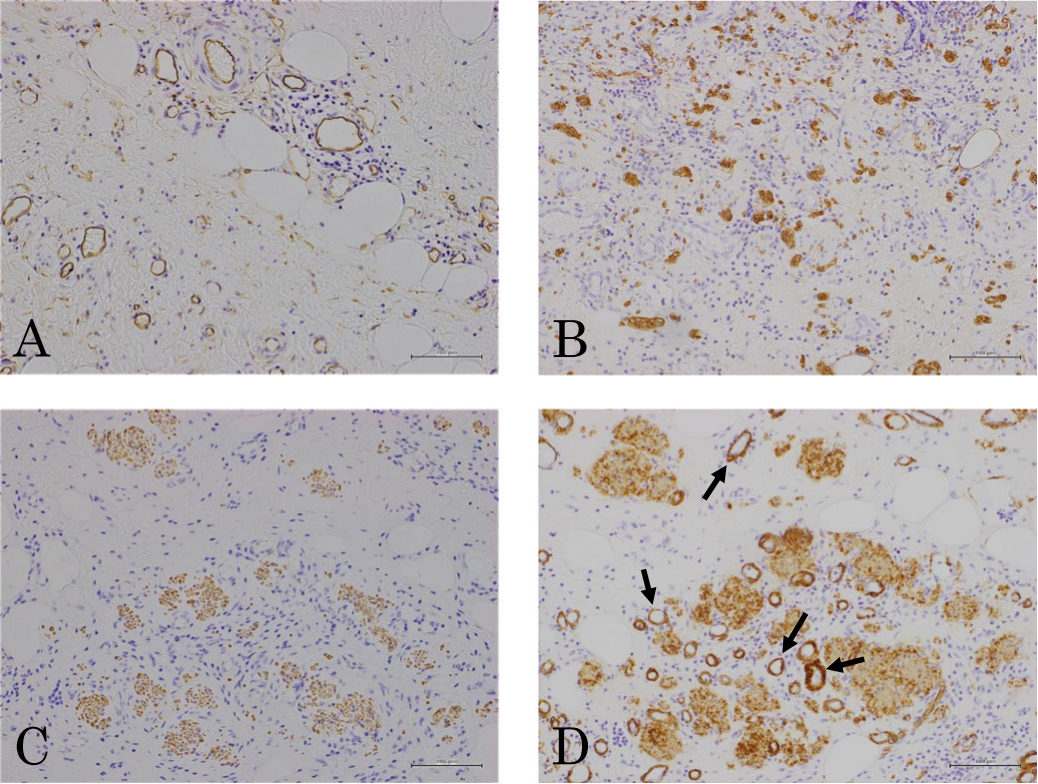
Immunohistochemical staining. Immunohistochemical staining revealed vascular endothelium (**a**: CD34), neural fibers (**b**: S-100), and smooth muscle fibers including perivascular fibers (arrows) (**c**: Desmin, **d**: smooth muscle actin), respectively. Immunoperoxidase staining. Original magnification, **a**: × 200; **b**, **c**, **d**: × 100

A 60-year-old man complaining of longstanding right lower quadrant pain for 4 years was referred to our hospital for further investigation and intervention. Abdominal examination revealed spontaneous pain in the lower right abdomen without rebound tenderness or palpation of a tumor. Blood examination showed no abnormal values including tumor markers. Abdominal computed tomography (CT) identified a slight swelling of the appendix (about 10 mm) without obvious neoplastic tumors or lymphadenopathy (Fig. 1). Colonoscopy showed elevation of the mucosa around the appendiceal orifice (Fig. 2). We considered the swollen appendix to be the cause of his pain and performed a laparoscopic appendectomy with partial resection of the cecum for diagnostic and therapeutic purposes. Intraoperative findings were a slight swelling of the appendix but no tumor exposure on the serosal surface of the appendix and cecum (Fig. 3). The surgical specimen contained a submucosal lesion located on the tip of the appendix that measured 18 × 10 mm (Fig. 4). Microscopically, this lesion was covered with normal mucosa, arose from submucosa, and contained abundant adipose tissue (Fig. 5). Smooth muscle fiber bundles, small vessels, and neural fiber bundles were collected and scattered irregularly in the submucosa, and there was no evidence of malignancy (Fig. 6). Each component of this lesion was confirmed by immunohistochemical examination. Vascular endothelium, neural fibers, and smooth muscle fibers were immunostained with CD34 (Fig. 7a), S-100 (Fig. 7b), and desmin (Fig. 7c)/smooth muscle actin (Fig. 7d) antibodies, respectively. These pathological findings were consistent with previous reports, and this lesion was diagnosed as NMVH. The patient’s right lower abdominal pain disappeared immediately after surgery, and was thought to be caused by the NMVH.

## Discussion

To date, 27 cases of NMVH have been reported, including the present case (Table 1). The mean patient age is 53.7 (12–91) years, and there are 10 males and 15 females, with age unknown in 4 cases and sex unknown in 2 cases. Most of the patients complained of abdominal pain, and 3 patients had Crohn’s disease as a comorbidity. NMVH originated in the small intestine in all cases except one in which it originated in the cecum [1–20]. Therefore, this report appears to be the first case of NMVH occurring in the appendix.

**Table 1:** Summary of 27 cases of neuromuscular and vascular hamartoma

	Author	Year	Age	sex	Symptom	Origin	Comorbidity
1	Fernando [1]	1982	30	Female	Anemia	Small intestine	
2	Fernando [1]		36	Female	Abdominal pain, vomiting	Small intestine	
3	Smith [2]	1986	51	Female	Abdominal pain, vomiting	Small intestine	
4	Shepherd [3]	1987	34	Female	Abdominal pain, vomiting	Small intestine	Crohn’s disease
5	Shepherd [3]		58	Male	Abdominal pain, diarrhea	Small intestine	Crohn’s disease
6	Shepherd [3]		73	Female	Abdominal pain	Small intestine	
7	Shepherd [3]		63	Female	Abdominal pain	Small intestine	
8	Kwasnik [4]	1989	91	Male	ND	Small intestine	
9	Salas [5]	1990	ND	ND	ND	Small intestine	
10	Cortina [6]	1999	73	Male	Bowel obstruction	Small intestine	
11	Cortina [6]		76	Male	Bowel obstruction	Small intestine	
12	De Sanctis [7]	2001	76	Female	Anemia	Small intestine	
13	Scintu [8]	2001	50	Male	Bowel obstruction	Small intestine	
14	Shiomi [9]	2002	76	Female	Asymptomatic	Cecum	
15	Company [10]	2005	76	Female	ND	Small intestine	
16	Theodosiou [11]	2009	60	Male	Abdominal pain	Small intestine	
17	Krishnamurthy [12]	2010	32	Male	Abdominal pain	Small intestine	
18	Kaplan [13]	2013	12	Female	Abdominal pain, vomiting	Small intestine	
19	Ren [14]	2014	ND	Female	Abdominal pain, anemia	Small intestine	
20	Ren [14]		ND	ND	ND	Small intestine	
21	Setaffy [15]	2015	ND	ND	ND	Small intestine	
22	Crothers [16]	2014	73	Female	ND	Small intestine	
23	Liu [17]	2015	27	Female	Abdominal pain, diarrhea	Small intestine	
24	Elster [18]	2016	59	Female	Abdominal pain, vomiting	Small intestine	Crohn’s disease
25	Caruso [19]	2018	58	Male	Abdominal pain, anemia	Small intestine	
26	Pattnaik [20]	2019	45	Male	ND	Small intestine	
27	Our case	2020	60	Male	Abdominal pain	Appendix	

*ND* not described

The only findings in this case were longstanding right lower quadrant pain and a slight swelling of the appendix on CT imaging. As a cause of the swollen appendix without inflammation, the latency of cancer and carcinoid tumors was considered. However, we presumed that the preoperative diagnosis was less likely to be a progressive disease such as cancer because no worsening of abdominal pain or tumor formation had been observed for the 4 years since onset. For these reasons, we performed a laparoscopic appendectomy for diagnostic and therapeutic purposes. The patient’s right lower abdominal pain disappeared immediately after surgery, indicating that it was likely caused by the NMVH. Although a detailed mechanism for the abdominal pain was unclear, a rise in the internal pressure of the appendix was considered as one possible cause.

NMVH has been thought to be a hamartoma that consists of an aberrant proliferation of neural, muscular, and vascular elements in the intestine [1]. However, Shepherd and Jass suggested that NMVH may represent an abnormal histologic consequence of chronic inflammatory bowel disease, especially Crohn’s disease [3]. Other authors reported that NMVH might not be a rare entity but rather a process of chronic inflammation [22, 23]. However, considering that most of the patients in the previous reports did not exhibit prominent fibrosis of the intestinal wall when observed in the chronic inflammatory state, it is thought that NMVH may exist as a separate entity [18]. For such reasons, the debate continues as to whether this lesion is truly a hamartoma or represents a “burnt-out” phase of various chronic pathologies.

The case presented here showed no histological features such prominent fibrosis that would indicate active or “burnt out” Crohn’s disease or another reactive process induced by chronic inflammation. Therefore, we consider this NMVH of the appendix to be an entity separate from any special state of chronic inflammation.

## Conclusion

We experienced a case of NMVH occurring in the appendix. Although NMVH is a benign disease, it should be included in the differential diagnosis of neoplastic lesions not only in the small intestine but also in the appendix.

## Data Availability

All data generated or analyzed during this study are included in this published article.

## Abbreviations

CT: Computed tomography
NMVH: Neuromuscular and vascular hamartoma
